# Myasthenia Gravis as a Manifestation of Aicardi–Goutières Syndrome Due to a SAMHD1 Variant Successfully Treated With Baricitinib

**DOI:** 10.7759/cureus.91485

**Published:** 2025-09-02

**Authors:** Maria Kollia, Stella Mouskou, Lida Mentesidou, Angeliki Syggelou, Katherine Anagnostopoulou, Despoina N Maritsi

**Affiliations:** 1 2nd Department of Pediatrics, ‘Panagiotis and Aglaia Kyriakou’ Children’s Hospital, National and Kapodistrian University of Athens, Athens, GRC; 2 Department of Neurology, ‘Panagiotis and Aglaia Kyriakou’ Children’s Hospital, National and Kapodistrian University of Athens, Athens, GRC; 3 1st Department of Pediatrics, ‘Panagiotis and Aglaia Kyriakou’ Children’s Hospital, National and Kapodistrian University of Athens, Athens, GRC; 4 Department of Molecular Genetics, Genomedica S.A., Piraeus, GRC

**Keywords:** aicardi-goutières syndrome, baricitinib, chilblains, encephalopathy, juvenile myasthenia gravis (jmg), myasthenia gravis (mg)

## Abstract

Aicardi-Goutières syndrome (AGS) is a rare inherited disorder that mainly affects the brain, immune system, and skin. It is caused by mutations that lead to calcium buildup in the brain, which is thought to trigger an immune response. Autoimmune features, such as chilblains, overlap with those seen in systemic lupus erythematosus. We describe a case of a male toddler with an AGS phenotype and a heterozygous mutation in the SAMHD1 gene, who developed juvenile myasthenia gravis (MG) and was successfully treated with baricitinib. To our knowledge, this is the first reported case of AGS associated with MG. The patient showed significant clinical improvement, including resolution of myasthenic symptoms and normalization of autoimmune markers over an 18-month follow-up period. This case highlights the importance of considering broader autoimmune manifestations in AGS and the potential efficacy of Janus kinase (JAK) inhibitors such as baricitinib.

## Introduction

Aicardi-Goutières syndrome (AGS) is a rare genetic condition (Online Mendelian Inheritance in Man (OMIM) #612952) that mostly affects the brain, immune system, and skin and is increasingly recognized for its association with autoimmune features. In this context, to the best of our knowledge, we report the first known case of AGS co-occurring with juvenile myasthenia gravis (MG), a neuromuscular autoimmune disorder that has not previously been linked to AGS. Although AGS is known for its immune dysregulation, MG has never been previously reported as part of the AGS clinical spectrum.

AGS belongs to a group of disorders known as type I interferonopathies, which are caused by an overactive immune response driven by excessive production of IFN-I. Children with AGS often present early in life with developmental delay, seizures, microcephaly, and characteristic brain imaging findings, including calcifications. Signs of immune system involvement may also occur, including unexplained fevers, chilblains, thyroid dysfunction, or lupus-like features [1-2]. AGS is genetically heterogeneous and is most often inherited in an autosomal recessive manner, involving mutations in genes like TREX1, RNASEH2A, RNASEH2B, RNASEH2C, ADAR1, and SAMHD1. [3] However, dominant mutations have also been identified, particularly in the IFIH1 gene. [4] Loss-of-function mutations in the SAMHD1 gene are associated with overproduction of IFN-I and account for 13% of AGS cases, and are associated with a wide range of clinical manifestations. Common features include chilblains (54.2%), glaucoma, non-destructive arthritis, demyelinating peripheral neuropathy, and gastrointestinal tract inflammation [5-7].

Juvenile MG is a rare autoimmune disease characterized by impaired neuromuscular transmission due to antibodies targeting the AChR. It leads to fluctuating muscle weakness, especially affecting the eyes, face, and throat.

In this case report, we present the case of a young child with AGS due to a heterozygous SAMHD1 variant who also developed juvenile MG. He responded favorably to treatment with baricitinib, a Janus kinase (JAK) inhibitor, suggesting that targeted IFN pathway modulation may be effective in this clinical scenario. We hope this case broadens the clinical understanding of AGS, highlights the possibility of consideration of autoimmune neuromuscular complications, and supports further exploration of targeted therapies like baricitinib.

## Case presentation

**Figure 1:**
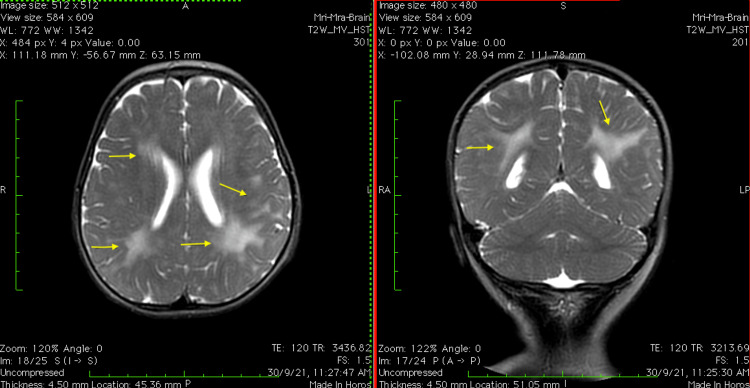
The patient's brain MRI Increased signal intensity on T2 and FLAIR and low signal intensity on T1 are seen in the periventricular white matter symmetrically, especially posteriorly, with extension to the subcortical white matter, especially parietally FLAIR: fluid-attenuated inversion recovery

A 20-month-old male toddler presented with bilateral blepharoptosis of five months’ duration. The patient was born full term, small for gestational age, via cesarean section. There was no parental consanguinity. From the age of four months, he developed acquired microcephaly, peripheral spasticity, and truncal hypotonia, as well as global developmental delay. Upon admission, clinical examination revealed a symmetrically small toddler (8 kg, 76 cm, 42 cm, all <3^rd^ percentile) with bilateral blepharoptosis, ophthalmoplegia, weak cry, dysphagia, truncal hypotonia, and limb hypertonia. He was developmentally capable of babbling, smiling at his mother, grasping objects, and rolling from a prone to a supine position. A brain MRI scan revealed leukoencephalopathy (Figure 1). Furthermore, he presented an annular erythema at the right inguinal area and a scar on the dorsal surface of the right foot, which were first noticed during winter, with features consistent with chilblains.

Full screening for inborn errors of metabolism, congenital infections, and mitochondrial diseases was negative. His karyotype was 46XY. However, laboratory tests were remarkable for mild elevation of liver enzymes, mild neutropenia, elevated ESR, and IgG levels. ANA, anti-dsDNA, and anti-TPO antibodies were positive, and thyroid-stimulating hormone (TSH) was high (42.3 μIU/ml). Concerning the screening for MG, AChR-Abs were positive (Table 1).

**Table 1:** The patient's laboratory test results

Parameter	Patient value	Reference range
Glucose	68 mg/dl	70-100
Blood urea nitrogen (BUN)	25 mg/dl	5-45
Creatinine	0.3 mg/dl	<0.7
Potassium	5.5 mEq/L	3.5-5.5
Sodium	137 mEq/L	134-148
Alkaline phosphatase (ALP)	189 U/l	<440
Aspartate aminotransferase (AST)/Alanine aminotransferase (ALT)	60/71 U/l	15-55/5-45
Gamma-glutamyl transferase (GGT)	7 U/l	<25
Creatine phosphokinase) (CPK)	54 U/l	<228
WBC	7,300/μl	6-17,000
Neutrophils (NE)/Lymphocytes (LY)	1,400/5,200	1,500-8,500/3,000-9,500
Hemoglobin (Hb)/Hematocrit (Hct)	14.4 gr/dl / 41.2%	9.5-14.5/33-39
Platelet count (Plts)	308,000/μl	130,000-400,000
ESR	45 mm	<15
CRP	0 mg/L	<5
IgG	1,880 mg/dl	575-1,446
IgA	<6.4 mg/dl	23-123
IgM	112mg/dl	63-251
ANA	1:160	Negative 1:80
Anti-dsDNA	Weakly positive	Negative
Anti-Ro/anti-La	Negative	Negative
Rheumatoid arthritis (RA) test	<10.1 U/ml	<30
C3/C4	127/24.4 mg/dl	90-180/13-75
Thyroid-stimulating hormone (TSH)	42 μIU/ml	0.5-6.46
Free thyroxine (FT4)	0.96 ng/dl	0.9-1.9
Anti-TPO	307.7 IU/ml	<16
Anti-TG	8.43 IU/ml	<100
Cortisol (morning)	13.66 μg/dl	6.2-23
Anti-musk	<0.015 nmol/L	Negative <0.015
AchR-Abs	12 nmol/L	Positive >0.6

**Figure 2:**
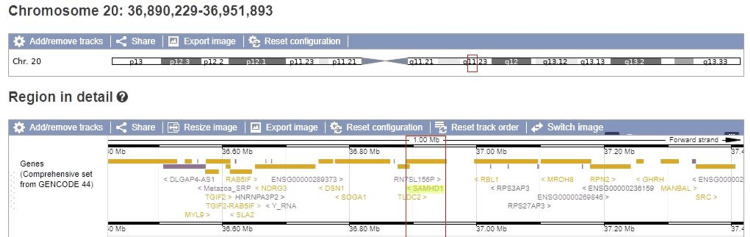
Whole exome sequencing (WES) Genomic location of the SAMHD1 gene and identification of a novel heterozygous variant (c.1351C>T, p.Arg451Cys) detected by WES. The upper panel shows the location of the SAMHD1 gene on chromosome 20q11.23. The lower panel presents a detailed view of the surrounding genomic region based on the GENCODE v44 gene annotation. The variant is mapped to transcript NM_015474.3. This variant results in an arginine-to-cysteine substitution at position 451 of the SAMHD1 protein.

Furthermore, edrophonium chloride (Tensilon) testing was positive, and upper limb electromyography and electroneurography findings were compatible with synaptic or postsynaptic neuromuscular dysfunction. Accordingly, the patient was treated with a short course of pyridostigmine, levothyroxine, and prednisolone (slow tapering). Finally, whole exome sequencing revealed a heterozygous variant c.1351C>T p.Arg451Cys on SAMHD1 (NM_015474.3, hg38) (Figure 2), confirmed by Sanger sequencing. Maternal testing was negative; the paternal sample was not available. The variant has a very low allele frequency (T: 0.0003%) in the gnomADin population database v4.1.0 (Broad Institute of Massachusetts Institute of Technology (MIT) and Harvard, Cambridge, MA). Computational prediction tools (Sorting Intolerant From Tolerant (SIFT), Likelihood Ratio Test (LRT), MutationTaster, FATHMM (FATHMM), Protein Variation Effect Analyzer (PROVEAN), and Meta Support Vector Machine (MetaSVM)) unanimously supported a deleterious effect on the gene. Based on the American College of Medical Genetics and Genomics (ACMG) variant classification guidelines [8] and on the latest ClinGen recommendations [9], the identified variant was classified as likely pathogenic (PP3_strong, PM2). The characteristic AGS phenotype of our patient meant that this variant on SAMHD1 could possibly support a dominant effect, although the known pathogenic variants so far are recessive. This observation expands the current understanding of the inheritance spectrum of SAMHD1-related AGS and has implications for both diagnosis and genetic counseling.

Subsequently, treatment with baricitinib, a JAK kinase inhibitor (1 mg/kg/day), was initiated, and it was well-tolerated. Follow-up at 18 months revealed resolution of MG and improvement in motor and developmental skills. The brain MRI/MR angiography (MRA) scan had no structural changes. Finally, ANA, anti-dsDNA, and AchR-Ab tests turned negative; however, ESR remained slightly elevated.

## Discussion

To the best of our knowledge, dominant variants in SAMHD1 associated with the AGS phenotype have not been previously reported. Herein, we report a heterozygous mutation associated with both neurological and autoimmune manifestations typical of AGS. One limitation of our study is the lack of measurement of cerebrospinal fluid IFN-I or expression of IFN-stimulated genes (ISGs).

Furthermore, juvenile MG has not been previously described as a neurological autoimmune manifestation in the context of AGS due to the SAMHD1 variant. This case, therefore, expands the phenotypic spectrum of AGS and underscores the possibility of overlap with antibody-mediated neuromuscular disorders.

Regarding the role of IFN in MG, treatment with IFN-α for chronic hepatitis C or IFN-α/IFN-β for multiple sclerosis is reported to induce myasthenic symptoms associated with AChR-Abs [10,11]. While IFN-I appears to be undetectable in the serum of AChR-MG patients, an IFN-I signature has been demonstrated in the thymus, and IFN-β is believed to contribute to thymic changes associated with early-onset AChR-positive MG [12]. 

In patients with AGS due to SAMHD1 variants, baricitinib, a JAK inhibitor, has shown promise, leading to regression of chilblains, decrease of ISG expression, and improvement in developmental milestones and skills [13]. In our case, an 18-month follow-up revealed remarkable clinical improvement, including resolution of MG symptoms and seronegativity for AChR-Abs and ANA. However, our patient experienced reappearance of chilblains during winter. A developmental improvement was also observed; however, we acknowledge that such progress may partly reflect the natural course of AGS, where the period of neurological damage appears to be limited to an initial encephalopathic phase and further progression is rather unusual in the classic AGS phenotype. Thus, attributing neurological gains solely to baricitinib requires caution. While our findings support the potential benefit of baricitinib in AGS, further studies are needed to confirm its long-term efficacy and safety, especially in pediatric populations. Alternative or adjunctive treatments, such as other JAK inhibitors or IFN-blocking antibodies, could also be considered in future cases.

## Conclusions

Our case highlights a heterozygous variant in SAMHD1 associated with a clinical phenotype of AGS and juvenile myasthenia gravis, expanding the known genetic and autoimmune spectrum of AGS. Our findings support the role of genetic analysis in the diagnostic evaluation of complex neuroimmunological presentations. Treatment with baricitinib led to the resolution of blepharoptosis, disappearance of AchR-Abs, and improved motor milestones, demonstrating its therapeutic potential. Further research is needed to elucidate the mechanisms linking IFN signaling with neuromuscular autoimmunity and to evaluate the efficacy of JAK inhibitors in larger cohorts.
